# Breast cancer risk in relation to history of preeclampsia and hyperemesis gravidarum: Prospective analysis in the Generations Study

**DOI:** 10.1002/ijc.31364

**Published:** 2018-03-23

**Authors:** Lauren B. Wright, Minouk J. Schoemaker, Michael E. Jones, Alan Ashworth, Anthony J. Swerdlow

**Affiliations:** ^1^ Division of Genetics and Epidemiology The Institute of Cancer Research London United Kingdom; ^2^ Division of Breast Cancer Research The Institute of Cancer Research London United Kingdom; ^3^ Breakthrough Breast Cancer Research Centre at the Institute of Cancer Research London United Kingdom; ^4^ Division of Molecular Pathology The Institute of Cancer Research London United Kingdom; ^5^ Present address: UCSF Helen Diller Family Comprehensive Cancer Center San Francisco CA 94158 USA

**Keywords:** breast cancer, cohort study, preeclampsia, hyperemesis gravidarum

## Abstract

Preeclampsia and hyperemesis gravidarum are pregnancy complications associated with altered sex hormone levels. Previous studies suggest preeclampsia may be associated with a decreased risk of subsequent breast cancer and hyperemesis with an increased risk, but the evidence remains unclear. We used data from the Generations Study, a large prospective study of women in the United Kingdom, to estimate relative risks of breast cancer in relation to a history of preeclampsia and hyperemesis using Cox regression adjusting for known breast cancer risk factors. During 7.5 years average follow‐up of 82,053 parous women, 1,969 were diagnosed with invasive or *in situ* breast cancer. Women who had experienced preeclampsia during pregnancy had a significantly decreased risk of premenopausal breast cancer (hazard ratio (HR) =0.67, 95% confidence interval (CI): 0.49–0.90) and of HER2‐enriched tumours (HR = 0.33, 95% CI: 0.12–0.91), but there was no association with overall (HR = 0.90, 95% CI: 0.80–1.02) or postmenopausal (HR = 0.97, 95% CI: 0.85–1.12) breast cancer risk. Risk reductions among premenopausal women were strongest within 20 years since the last pregnancy with preeclampsia. Hyperemesis was associated with a significantly increased risk of HER2‐enriched tumours (HR = 1.76, 95% CI: 1.07–2.87), but not with other intrinsic subtypes or breast cancer risk overall. These results provide evidence that preeclampsia is associated with a decreased risk of premenopausal and HER2‐enriched breast cancer and that hyperemesis, although not associated with breast cancer risk overall, may be associated with raised risk of HER2‐enriched tumours.

## Abstract 2

**What's new?**

Although the link of preeclampsia and hyperemesis gravidarum with altered sex hormone levels is well established, relatively little is known about the association of these pregnancy complications with breast cancer risk. Here, the authors found that a history of preeclampsia decreased the risk of premenopausal breast cancer and tumors enriched for the receptor tyrosine‐protein kinase HER2. In contrast, hyperemesis gravidarum increased the risk to develop HER2‐enriched tumors, pointing to nuanced differences of sex hormone alterations during pregnancy with respect to breast cancer subtypes and menopausal status.

**Abbreviations**
BMI: body mass index
CI: confidence interval
ER: oestrogen receptor
HER2: human epidermal growth factor receptor 2
HR: hazard ratio
UK: United Kingdom

## Introduction

Breast cancer is the most common type of cancer among females worldwide and has a hormonal aetiology. During pregnancy, breast tissue is exposed to hormonal surges including of oestrogen and progesterone, which may be related to the transient increased risk of breast cancer after pregnancy, followed by a decreased risk long‐term.^1^, ^2^ Disorders that affect hormone levels in pregnancy might therefore also affect subsequent breast cancer risk.

Preeclampsia is a disorder characterised by the onset of hypertension and proteinuria in the second half of pregnancy, affecting around 2–8% of pregnancies,^3^ which is associated with decreased levels of estradiol^4^ and insulin‐like growth factors.^5^, ^6^ It has been hypothesised that preeclampsia might therefore be associated with a decreased risk of maternal breast cancer.^7^ Evidence from previous studies has been inconsistent, however, with studies reporting negative,^7^, ^8^, ^9^, ^10^, ^11^ positive^12^ or no^13^, ^14^, ^15^, ^16^, ^17^ association with breast cancer risk. Few studies have been of prospective design and the studies have often not been able to adjust for potential confounders. Only a few studies have reported on whether the association depends on age at birth,^9^, ^18^ number of preeclamptic pregnancies,^10^ sex of offspring,^8^, ^18^ menopausal status^10^ or invasiveness of breast cancer^13^ and none have investigated the association by breast cancer intrinsic subtype.

Hyperemesis gravidarum (hereafter referred to as ‘hyperemesis’) is characterised by severe nausea, vomiting and faintness in the first half of pregnancy. It has been hypothesised that it could be associated with a raised risk of maternal breast cancer via elevated estradiol levels in pregnancy.^19^ The few past studies have not consistently shown an association of hyperemesis with breast cancer risk,^13^, ^14^, ^20^, ^21^, ^22^, ^23^, ^24^ but all except one^13^ have been of case–control design, which could be prone to recall bias.

We therefore examined the association between history of preeclampsia and hyperemesis and maternal risk of breast cancer in women within the Generations Study, a large prospective cohort study in the UK, in which detailed information on pregnancies, breast cancer risk factors and breast cancer characteristics was available.

## Materials and Methods

The Generations Study is a cohort study of over 113,700 women from the United Kingdom (UK), aged 16 years or older, designed to investigate the aetiology of breast cancer. Questionnaire information and informed consent were gained at recruitment since 2003. The first follow‐up questionnaire (2½ years after recruitment) was completed by 99% of non‐deceased participants, the second (6 years after recruitment) by 96%, and the third (9½ years after recruitment) by 94% of those recruited long enough ago to have entered this round of follow‐up. The study was approved by the South East Multi‐Centre Research Ethics Committee and has been described in detail previously.^25^

Breast cancers occurring in the cohort were identified from recruitment and follow‐up questionnaires and spontaneous reports to the study centre, and where necessary from ‘flagging’ at the National Health Service Central Registers (virtually complete register of the populations of England, Wales and Scotland, to which study participants can be linked and on which deaths and cancer registrations are ‘flagged’ and then reported to authorised medical researchers). Confirmation of diagnosis was obtained from UK cancer registries, pathology reports and correspondence with patients' general practitioners.

Information on pregnancies was collected in the recruitment and second follow‐up questionnaires. Participants were asked if they had experienced preeclampsia or hyperemesis gravidarum (i.e., severe vomiting every day for at least a week in the first 3 months of pregnancy), and about birth outcome, gestational length, offspring sex and birth weight at each of their pregnancies. Age at first and last pregnancy and time since last pregnancy with preeclampsia and hyperemesis were calculated using information from individual pregnancies.

Information on menopausal status and age at menopause was obtained from the recruitment and second follow‐up questionnaires, in which women were asked how old they were when their periods stopped completely (i.e., they had gone 6 months without having had a period). For women who did not provide an age at menopause but who reported that they became postmenopausal during follow‐up, we defined menopausal age to be the youngest of either the woman's age at the questionnaire at which she first reported that she was postmenopausal or age 50.

### Statistical analysis

The analytic cohort in our study is based on all women who were recruited to the Generations Study before 31 December 2013, did not have a previous diagnosis of invasive or *in situ* breast cancer, and were either parous (defined as pregnancies of ≥26 weeks resulting in a live birth) at recruitment or became parous during follow‐up. The recruitment cut‐off at December 2013 was selected because at the time of analysis the second follow‐up was virtually complete, two‐thirds had reached the third follow‐up, and we had ‘flagging’ information to June 2017 for these recruits. Participants entered risk at the date of return of their recruitment questionnaire, or if they were nulliparous at recruitment and became parous during follow‐up, the date of first delivery, and were censored at the earliest of: first invasive or *in situ* breast cancer; death; most recent follow‐up questionnaire (depending on date of recruitment), if completed, or the date the most recent follow‐up questionnaire was due if cancer and vital status was known from ‘flagging’; or the date of the last completed questionnaire, if lost to study follow‐up. We additionally censored at the first age of having a twin or higher‐order birth, since such births are associated with higher hormone exposures.^26^ Cox proportional hazards regression^27^ using attained age as the implicit time scale was used to estimate hazard ratios (HR) and 95% confidence intervals (CI) for risk of first breast cancer in relation to pregnancy complications. Models were adjusted for: socio‐economic score;^28^ birth cohort; benign breast disease; family history of breast cancer in 1st degree relatives; age at menarche; parity; age at first birth; number of parous births; duration of breastfeeding across all births; body mass index (BMI) at age 20 years; postmenopausal BMI, if applicable; height at recruitment; premenopausal oral contraceptive use; menopausal status; age at menopause; hormone replacement therapy use; alcohol consumption; cigarette smoking status and physical activity level. The baseline reference group for pregnancy complication variables was comprised of parous women with no history of preeclampsia, or of hyperemesis, depending on the risk factor under analysis. Pregnancy and complications of pregnancy data were analysed as time‐dependent variables, such that women were considered as having no history of preeclampsia before a pregnancy with the condition, and then contributed to the preeclampsia risk group from that pregnancy onwards. HRs were estimated for breast cancer overall and subdivided by time‐updated menopausal status, oestrogen receptor (ER) status, invasiveness and intrinsic subtype of breast cancer. Breast cancer intrinsic subtype definitions were adapted from those proposed by the St Gallen Expert Consensus.^29^ To assess the trends in risk across number of pregnancies and time since last pregnancy with complications we modelled continuous variables, excluding women with zero pregnancies. Likelihood ratio tests^30^ were used to test for interactions between preeclampsia variables and the subjects' mother's history of preeclampsia, sex of the offspring at first and last pregnancy with preeclampsia, and between history of preeclampsia and history of hyperemesis. All statistical tests were two‐sided and analyses were conducted using Stata/IC version 14.2.^31^

## Results

During 2003–2013 the recruitment questionnaire was completed by 106,628 study participants who had no previous invasive or *in situ* breast cancer, or prophylactic mastectomy. Women who remained nulliparous throughout follow‐up (*n* = 22,359) and women who had a twin or higher‐order birth before entry to the study (*n* = 2,216) were excluded from the analysis. The analytical cohort included 82,053 women who were either parous at baseline questionnaire (*n* = 78,539) or became parous during follow‐up (*n* = 3,514) and therefore entered follow‐up at their first parous birth. Three hundred and twenty nine women were censored at their first twin or higher‐order birth during follow‐up. During follow‐up, 1.2% of women died. Of the remainder, cancer and vital status was known for 97.4% from completion of the relevant follow‐up questionnaire, and a further 2.4% from ‘flagging’ at the National Health Service Central Registers. The remaining 0.2% were lost to follow‐up at an earlier date. The follow‐up completeness (calculated as the total observed person‐years divided by the maximum person‐years that would have been achievable if all women were followed‐up to their relevant questionnaire or, if earlier, death) was 99.7%. There were 1,969 incident breast cancers during follow‐up (1,665 invasive, 304 *in situ*), of which 99.6% were confirmed through national cancer registration or medical records and the remaining eight cases were self‐reported with treatments that implied breast cancer. ER information was available for 91.2% of breast cancers (99.3% of invasive, 47.0% of *in situ*), and of these 83% were ER positive.

The median age at recruitment was 49.0 years (inter‐quartile range: 39–58) and participants contributed an average of 7.5 years of follow‐up. Characteristics of the participants overall and by preeclampsia and hyperemesis history are given in Table 1, 28. In the analytical cohort, 99% of women self‐reported as Caucasian, 14.8% reported that they had experienced preeclampsia during at least one of their full‐term pregnancies, whilst 28.7% reported hyperemesis.

**Table 1:** Characteristics of the analytical cohort

Characteristic	All		Ever preeclampsia^a^		Ever hyperemesis gravidarum^a^	
	N subjects	%	N subjects	%	N subjects	%
*Age at start of follow‐up, years*						
<30	3,519	4.3	316	2.6	923	3.9
30–39	18,282	22.3	1,874	15.4	4,013	17.1
40–49	19,688	24.0	2,842	23.4	5,235	22.3
≥50	40,564	49.4	7,104	58.5	13,345	56.7
*Number of births*						
1	20,140	24.5	2,166	17.8	3,992	17.0
2	41,925	51.1	6,522	53.7	12,643	53.8
≥3	19,988	24.4	3,448	28.4	6,881	29.3
*Age at first birth, years*						
≤24	25,519	31.1	4,540	37.4	10,527	44.8
25–34	50,395	61.4	6,946	57.2	12,190	51.8
≥35	6,124	7.5	648	5.3	795	3.4
Parous, unknown age	15	0.02	3	0.02	4	0.02
*Preeclampsia*						
Never	69,917	85.2	–	–	19,183	81.6
Ever	12,136	14.8	12,136	100.0	4,333	18.4
*Number of pregnancies with preeclampsia*						
1	8,686	71.6	8,686	71.6	2,915	12.4
2	2,756	22.7	2,756	22.7	1,105	4.7
≥3	694	5.7	694	5.7	313	1.3
*Hyperemesis gravidarum*						
Never	58,537	71.3	7,803	64.3	–	–
Ever	23,516	28.7	4,333	35.7	23,516	100.0
*Number of pregnancies with hyperemesis*						
1	10,532	44.8	1,816	15.0	10,532	44.8
2	9,068	38.6	1,744	14.4	9,068	38.6
≥3	3,916	16.7	773	6.4	3,916	16.7
*Menopausal status at start of follow‐up*						
Premenopausal	42,113	51.3	5,114	42.1	10,180	43.3
Postmenopausal	35,636	43.4	6,263	51.6	11,909	50.6
Unknown	4,304	5.2	759	6.3	1,427	6.1
*First‐degree family history of breast cancer at recruitment*						
No	69,092	84.2	10,205	84.1	19,604	83.4
Yes	12,961	15.8	1,931	15.9	3,912	16.6
*BMI at recruitment, units (kg/m* b *)*						
<20	4,551	5.5	378	3.1	978	4.2
20–24.9	39,008	47.5	4,476	36.9	9,855	41.9
≥25	35,625	43.4	6,937	57.2	11,953	50.8
Unknown	2,869	3.5	345	2.8	730	3.1
*Socio‐economic status based on place of residence at recruitment* b						
1 (highest)	39,603	48.3	5,919	48.8	11,221	47.7
2	7,718	9.4	858	7.1	1,643	7.0
3	23,198	28.3	3,527	29.1	6,851	29.1
4	6,314	7.7	1,023	8.4	1,998	8.5
5 (lowest)	4,581	5.6	732	6.0	1,630	6.9
No classification	639^c^	0.8	77	0.6	173	0.7
*Total subjects*	82,053	100.0	12,136	100.0	23,516	100.0

a Self‐reported history of preeclampsia and hyperemesis gravidarum.

b Based on ACORN definitions of place of residence.^28^

c No classification for 414 because they were resident in the Channel Islands for which ACORN coding is not available; and for 225 for other reasons e.g. resident in an institution.

### Preeclampsia

Women who reported a history of preeclampsia during a pregnancy had a similar risk of breast cancer overall compared to parous women who had never experienced preeclampsia (HR = 0.90, 95% CI: 0.80–1.02) (Table 2). Risk of breast cancer was significantly decreased during the 20 years after preeclampsia (HR = 0.75, 95% CI: 0.60–0.93), but 20 or more years after experiencing it, women had a similar risk to those who had never had preeclampsia. Risk was similarly reduced for preeclampsia at first pregnancy (HR = 0.86, 95% CI: 0.75–0.99) and most recent (hereafter referred to as ‘last’) pregnancy (HR = 0.88, 95% CI: 0.75–1.04). For premenopausal breast cancer a significant reduction in risk after preeclampsia was observed (HR = 0.67, 95% CI: 0.49–0.90) and in women with a history of one preeclamptic pregnancy (HR = 0.68, 95% CI: 0.49–0.95), but with no significant trend (*p*_trend_ = 0.29) across the number of pregnancies with preeclampsia. Risk reductions for premenopausal women were significant for women whose first preeclamptic pregnancy was at ages 25 or over (HR = 0.61, 95% CI: 0.43–0.86), or whose last preeclamptic pregnancy was at ages under 30 years (HR = 0.60, 95% CI: 0.39–0.92), or during the 20 years after the last preeclamptic pregnancy (HR = 0.64, 95% CI: 0.47–0.88). The risk of premenopausal breast cancer was also significantly reduced for preeclampsia experienced at a first (HR = 0.70, 95% CI: 0.51–0.97) or a last pregnancy (HR = 0.49, 95% CI: 0.31–0.79). The relative risk of premenopausal breast cancer in relation to a history of preeclampsia remained significantly decreased when the analyses were restricted to invasive breast tumours only (Supporting Information Table S1). For postmenopausal breast cancer, there was no significant association of risk with any of the above preeclampsia variables.

**Table 2:** Relative risk of breast cancer in relation to history of preeclampsia, by menopausal status

Risk factor			Menopausal status of subject at breast cancer incidence			
	Overall breast cancer		Premenopausal		Postmenopausal	
	N cases	Adjusted HR^a^ (95% CI)	N cases	Adjusted HR^b^ (95% CI)	N cases	Adjusted HR^a^ (95% CI)
*Never/ever preeclampsia*						
Never	1,673	1.00 (ref)	480	1.00 (ref)	1,193	1.00 (ref)
Ever	294	0.90 (0.80–1.02)	48	0.67 (0.49–0.90)	246	0.97 (0.85–1.12)
Number of pregnancies with preeclampsia*						
1	212	0.94 (0.82–1.09)	37	0.68 (0.49–0.95)	175	1.03 (0.88–1.20)
2	64	0.79 (0.61–1.01)	11	0.74 (0.41–1.35)	53	0.81 (0.61–1.06)
≥3	18	0.91 (0.57–1.46)	0	–	18	1.08 (0.68–1.74)
*p* trend^c^		*0.22*		*0.29*		*0.38*
Age at first preeclamptic pregnancy, years*						
<25	85	0.86 (0.68–1.08)	12	0.83 (0.45–1.51)	73	0.86 (0.67–1.11)
≥25	208	0.92 (0.79–1.06)	35	0.61 (0.43–0.86)	173	1.03 (0.87–1.21)
Age at most recent preeclamptic pregnancy, years*						
<30	174	0.86 (0.73–1.01)	22	0.60 (0.39–0.92)	152	0.92 (0.78–1.09)
≥30	119	0.96 (0.80–1.16)	25	0.71 (0.47–1.06)	94	1.07 (0.87–1.33)
Time since most recent preeclamptic pregnancy, years*						
<20	83	0.75 (0.60–0.93)	43	0.64 (0.47–0.88)	40	0.90 (0.65–1.24)
≥20	211	0.98 (0.85–1.14)	5	0.95 (0.38–2.35)	206	0.99 (0.85–1.15)
*p* trend^d^		*0.99*		*0.44*		*0.81*
*Preeclampsia at first pregnancy*						
No	1,738	1.00 (ref)	486	1.00 (ref)	1,252	1.00 (ref)
Yes	229	0.86 (0.75–0.99)	42	0.70 (0.51–0.97)	187	0.91 (0.78–1.06)
*Preeclampsia at last pregnancy*						
No	1,812	1.00 (ref)	510	1.00 (ref)	1,302	1.00 (ref)
Yes	155	0.88 (0.75–1.04)	18	0.49 (0.31–0.79)	137	0.99 (0.83–1.18)

HR: hazard ratio; CI: confidence interval.

*Reference group = never preeclampsia.

a Adjusted for attained age, socio‐economic score, birth cohort, benign breast disease, family history of breast cancer, age at menarche, age at first birth, number of births, duration of breast feeding, menopausal status, age at menopause, BMI at age 20 years, postmenopausal BMI, height, OC use, HRT use, alcohol consumption (units/wk), cigarette smoking status, physical activity level (METs/wk).

b Same as (1) but with removal of adjustment for menopausal status, age at menopause and postmenopausal BMI.

c Test for linear trend per pregnancy with preeclampsia, excluding zero pregnancies.

d Test for linear trend per 5 years since last preeclamptic pregnancy.

Relative risks with respect to preeclampsia were similar for ER positive and ER negative tumours (Table 3). Risk of ER positive breast cancer was significantly reduced within 20 years of the last preeclamptic pregnancy (HR = 0.76; 95% CI: 0.59–0.98), but not significantly associated with other variables. When analysed by invasiveness of breast cancer, risk reductions for invasive cancer were in general slightly greater than for breast cancer overall, with similar relative risks for first or last preeclamptic pregnancy at ages under 25 and 30 years respectively (HR = 0.72, 95% CI: 0.55–0.94; HR = 0.79; 95% CI: 0.66–0.95). For *in situ* breast cancer, however, there was no association with ever having had preeclampsia (HR = 1.01, 95% CI: 0.74–1.37), nor with subdivisions of this, except for a positive association in women aged under 25 years at their first preeclamptic pregnancy (HR = 1.62; 95% CI: 1.03–2.57). Ever having had preeclampsia was associated with a significantly decreased risk of human epidermal growth factor receptor 2 (HER2)‐enriched breast cancer (HR = 0.33; 95% CI: 0.12–0.91), but there was no association with other intrinsic subtypes (Table 4).

**Table 3:** Relative risk of breast cancer in relation to history of preeclampsia, by oestrogen receptor status and invasive/*in situ* breast cancer

Risk factor	ER status				Invasive/*in situ*			
	ER positive		ER negative		Invasive		*In situ*	
	N cases	Adjusted HR^a^ (95% CI)	N cases	Adjusted HR^a^ (95% CI)	N cases	Adjusted HR^a^ (95% CI)	N cases	Adjusted HR^a^ (95% CI)
*Never/ever preeclampsia*								
Never	1,266	1.00 (ref)	263	1.00 (ref)	1,419	1.00 (ref)	254	1.00 (ref)
Ever	225	0.90 (0.78–1.04)	40	0.80 (0.57–1.12)	244	0.88 (0.77–1.01)	50	1.01 (0.74–1.37)
Number of pregnancies with preeclampsia*								
1	158	0.92 (0.78–1.09)	30	0.86 (0.59–1.26)	171	0.90 (0.76–1.05)	41	1.20 (0.86–1.67)
2	53	0.85 (0.64–1.12)	6	0.49 (0.22–1.11)	56	0.81 (0.62–1.06)	8	0.65 (0.32–1.33)
≥3	14	0.93 (0.54–1.58)	4	1.36 (0.49–3.71)	17	1.03 (0.63–1.67)	1	0.31 (0.04–2.22)
*p* trend^b^		*0.45*		*0.74*		*0.63*		*0.06*
Age at first preeclamptic pregnancy, years*								
<25	60	0.81 (0.62–1.06)	14	0.82 (0.47–1.45)	61	0.72 (0.55–0.94)	24	1.62 (1.03–2.57)
≥25	165	0.94 (0.80–1.11)	26	0.79 (0.52–1.19)	183	0.95 (0.81–1.11)	25	0.72 (0.48–1.10)
Age at most recent preeclamptic pregnancy, years*								
<30	128	0.83 (0.69–1.00)	25	0.78 (0.51–1.18)	136	0.79 (0.66–0.95)	38	1.23 (0.87–1.74)
≥30	97	1.02 (0.83–1.26)	15	0.83 (0.49–1.41)	108	1.03 (0.84–1.25)	11	0.59 (0.32–1.09)
Time since most recent preeclamptic pregnancy, years*								
<20	63	0.76 (0.59–0.98)	12	0.66 (0.37–1.19)	67	0.72 (0.56–0.93)	16	0.87 (0.52–1.46)
≥20	162	0.98 (0.83–1.16)	28	0.88 (0.59–1.32)	177	0.97 (0.82–1.13)	34	1.09 (0.75–1.58)
*p* trend^c^		*0.92*		*0.86*		*0.80*		*0.84*
*Preeclampsia at first pregnancy*								
No	1,318	1.00 (ref)	269	1.00 (ref)	1,474	1.00 (ref)	264	1.00 (ref)
Yes	173	0.85 (0.73–1.00)	34	0.84 (0.59–1.21)	189	0.84 (0.72–0.98)	40	0.99 (0.71–1.39)
*Preeclampsia at last pregnancy*								
No	1,370	1.00 (ref)	284	1.00 (ref)	1,531	1.00 (ref)	281	1.00 (ref)
Yes	121	0.90 (0.75–1.09)	19	0.69 (0.43–1.11)	132	0.89 (0.74–1.06)	23	0.85 (0.56–1.31)

ER: oestrogen receptor; HR: hazard ratio; CI: confidence interval.

*Reference group = never preeclampsia.

a Adjusted for attained age, socio‐economic score, birth cohort, benign breast disease, family history of breast cancer, age at menarche, age at first birth, number of births, duration of breast feeding, menopausal status, age at menopause, BMI at age 20 years, postmenopausal BMI, height, OC use, HRT use, alcohol consumption (units/wk), cigarette smoking status, physical activity level (METs/wk).

b Test for linear trend per pregnancy with preeclampsia, excluding zero pregnancies.

c Test for linear trend per 5 years since last preeclamptic pregnancy.

**Table 4:** Relative risk of breast cancer by tumour intrinsic subtype in relation to history of preeclampsia and hyperemesis

Risk factor and intrinsic subtype of breast cancer^1^	Number of cases		Adjusted HR^2^ (95% CI)
	Ever	Never	
*Ever had preeclampsia*			
Luminal A‐like	91	483	0.97 (0.77–1.21)
Luminal B‐like	37	246	0.80 (0.57–1.14)
Luminal B‐like, HER2−	18	95	0.97 (0.58–1.61)
Luminal B‐like, HER2+	15	127	0.66 (0.39–1.13)
Luminal, NS	96	531	0.90 (0.72–1.12)
Non‐luminal	31	206	0.79 (0.54–1.15)
HER2‐enriched	4	66	0.33 (0.12–0.91)
Triple negative	23	108	1.08 (0.68–1.70)
*Ever had hyperemesis gravidarum*			
Luminal A‐like	168	406	0.97 (0.80–1.17)
Luminal B‐like	85	198	1.03 (0.79–1.34)
Luminal B‐like, HER2−	39	74	1.22 (0.82–1.82)
Luminal B‐like, HER2+	39	103	0.96 (0.66–1.41)
Luminal, NS	211	416	1.08 (0.91–1.28)
Non‐luminal	73	164	1.06 (0.80–1.41)
HER2‐enriched	28	42	1.76 (1.07–2.87)
Triple negative	40	91	1.00 (0.68–1.46)

ER: oestrogen receptor; PR: progesterone receptor; HER2: human epidermal growth factor 2; +: positive; −: negative; ?: not known; NS: not‐specified; RR: relative risk; CI: confidence interval.

^1^Intrinsic subtype groups are not mutually exclusive:

Luminal A‐like =ER + PR + HER2−.

Luminal B‐like = ER+/PR+ and HER2+; ER + PR− and HER2? ER− PR+ and HER2?

Luminal B‐like, HER2−=ER + PR− HER2−; ER− PR + HER2−.

Luminal B‐like, HER2+= ER+/PR+ and HER2+.

Luminal, NS = ER+/PR+ and HER2−; ER+/PR+ and HER2?

Non‐luminal = ER−PR−HER2?

HER2 enriched = ER−PR−HER2+.

Triple negative = ER−PR−HER2−.

^2^Adjusted for attained age, socio‐economic score, birth cohort, benign breast disease, family history of breast cancer, age at menarche, age at first birth, number of births, duration of breast feeding, menopausal status, age at menopause, BMI at age 20 years, postmenopausal BMI, height, OC use, HRT use, alcohol consumption (units/wk), cigarette smoking status, physical activity level (METs/wk).

Relative risks with respect to preeclampsia did not vary appreciably according to whether the subject's mother had experienced preeclampsia at the subject's birth (Table 5). The relative risk of breast cancer after preeclampsia during a first pregnancy was significantly reduced in women who gave birth to a girl (HR = 0.79; 95% CI: 0.65–0.97), but not in those who gave birth to a boy (HR = 0.94, 95% CI: 0.78–1.14), and no greater reduction for female than male offspring at the last birth, nor a significant interaction with sex of the offspring (*p*_int_ = 0.23 and *p*_int_ = 0.67). Relative risks with respect to preeclampsia did not vary significantly according to whether the subject also had a history of hyperemesis, or vice versa (*p*_int_ = 0.21) (Supporting Information Tables S2 and S3).

**Table 5:** Relative risk of breast cancer in relation to history of preeclampsia, mother's history of preeclampsia and sex of offspring

Risk factor	Subjects' mother had preeclampsia at subjects' pregnancy				*p*_int_ b	Sex of offspring at subjects' preeclampsia delivery				*p*_int_ b
	Yes		No			Boy		Girl		
	N cases	Adjusted HR^a^ (95% CI)	N cases	Adjusted HR^a^ (95% CI)		N cases	Adjusted HR^a^ (95% CI)	N cases	Adjusted HR^a^ (95% CI)	
*Never/ever preeclampsia*										
Never	43	1.00 (ref)	1,260	1.00 (ref)		*–*	*–*	*–*	*–*	*–*
Ever	20	0.85 (0.50–1.45)	182	0.93 (0.80–1.09)	0.75	*–*	*–*	*–*	*–*	*–*
*Number of pregnancies with preeclampsia*										
1	12	1.00 (ref)	136	1.00 (ref)		–	–	–	–	–
2	6	0.81 (0.30–2.20)	37	0.77 (0.53–1.12)		–	–	–	–	–
≥3	2	1.55 (0.34–7.11)	9	0.85 (0.42–1.75)	0.80	–	–	–	–	–
*Preeclampsia at first pregnancy*										
No	47	1.00 (ref)	1,299	1.00 (ref)		831	1.00 (ref)	904	1.00 (ref)	
Yes	16	0.79 (0.45–1.39)	143	0.90 (0.76–1.07)	0.65	123	0.94 (0.78–1.14)	106	0.79 (0.65–0.97)	0.23
*Preeclampsia at last pregnancy*										
No	51	1.00 (ref)	1,350	1.00 (ref)		883	1.00 (ref)	925	1.00 (ref)	
Yes	12	0.84 (0.45–1.57)	92	0.92 (0.74–1.13)	0.79	73	0.85 (0.67–1.08)	82	0.91 (0.73–1.15)	0.67

a Adjusted for attained age, socio‐economic score, birth cohort, benign breast disease, family history of breast cancer, age at menarche, age at first birth, number of births, duration of breast feeding, menopausal status, age at menopause, BMI at age 20 years, postmenopausal BMI, height, OC use, HRT use, alcohol consumption (units/wk), cigarette smoking status, physical activity level (METs/wk).

b Interaction test *p*‐values.

### Hyperemesis gravidarum

The overall risk of breast cancer in relation to ever having experienced hyperemesis was close to unity (HR = 1.03; 95% CI: 0.94–1.14) (Table 6). There was no trend of risk with number of pregnancies with hyperemesis (*p*_trend_ = 0.44) or time since last hyperemetic pregnancy (*p*_trend_ = 0.83), and no association with the age at hyperemetic pregnancy or with hyperemesis at first or last pregnancy. Relative risks did not vary by menopausal status at follow‐up, or ER status or invasiveness of breast cancer (Supporting Information Table S4). Risk of HER2‐enriched breast cancer was significantly raised in women who had experienced hyperemesis (HR = 1.76; 95% CI: 1.07–2.87), but there was no association with other intrinsic subtypes of breast cancer (Table 4).

**Table 6:** Relative risk of breast cancer in relation to history of hyperemesis gravidarum, by menopausal status

Risk factor			Menopausal status of subject at breast cancer incidence			
	Overall breast cancer		Premenopausal		Postmenopausal	
	N cases	Adjusted HR (95% CI)^a^	N cases	Adjusted HR (95% CI)^b^	N cases	Adjusted HR (95% CI)^a^
*Never/ever hyperemesis*						
Never	1,350	1.00 (ref)	386	1.00 (ref)	964	1.00 (ref)
Ever	617	1.03 (0.94–1.14)	142	1.10 (0.91–1.34)	475	1.01 (0.90–1.13)
Number of pregnancies with hyperemesis*						
1	240	1.00 (0.87–1.15)	64	1.14 (0.87–1.49)	176	0.96 (0.82–1.13)
2	274	1.09 (0.95–1.25)	61	1.13 (0.85–1.49)	213	1.08 (0.92–1.26)
≥3	103	0.96 (0.77–1.20)	17	0.91 (0.54–1.52)	86	0.97 (0.76–1.23)
*p* trend^c^		*0.44*		*0.34*		*0.62*
Age at first hyperemetic pregnancy, years*						
<25	266	1.07 (0.91–1.26)	35	1.12 (0.74–1.70)	231	1.06 (0.90–1.26)
≥25	351	1.01 (0.90–1.14)	107	1.10 (0.88–1.36)	244	0.98 (0.84–1.13)
Age at most recent hyperemetic pregnancy, years*						
<30	377	1.02 (0.91–1.15)	67	1.23 (0.93–1.62)	310	0.99 (0.86–1.13)
≥30	240	1.05 (0.91–1.20)	75	1.02 (0.79–1.30)	165	1.06 (0.90–1.26)
Time since most recent hyperemetic pregnancy, years*						
<20	197	1.06 (0.90–1.24)	131	1.10 (0.90–1.34)	66	0.99 (0.76–1.29)
≥20	420	1.02 (0.90–1.15)	11	1.14 (0.60–2.18)	409	1.02 (0.90–1.15)
*p* trend^d^		*0.83*		*0.80*		*0.56*
*Hyperemesis at first pregnancy*						
No	1,413	1.00 (ref)	403	1.00 (ref)	1,010	1.00 (ref)
Yes	554	1.04 (0.94–1.15)	125	1.14 (0.93–1.39)	429	1.01 (0.90–1.14)
*Hyperemesis at last pregnancy*						
No	1,509	1.00 (ref)	424	1.00 (ref)	1,085	1.00 (ref)
Yes	458	1.01 (0.90–1.12)	104	1.00 (0.80–1.24)	354	1.01 (0.89–1.14)

HR: hazard ratio; CI: confidence interval.

*Reference group = never hyperemesis.

a Adjusted for attained age, socio‐economic score, birth cohort, benign breast disease, family history of breast cancer, age at menarche, age at first birth, number of births, duration of breast feeding, menopausal status, age at menopause, BMI at age 20 years, postmenopausal BMI, height, OC use, HRT use, alcohol consumption (units/wk), cigarette smoking status, physical activity level (METs/wk).

b Same as (1) but with removal of adjustment for postmenopausal BMI.

c Test for linear trend per pregnancy with preeclampsia, excluding zero pregnancies.

d Test for linear trend per 5 years since last hyperemetic pregnancy.

## Discussion

In this large prospective investigation, women who experienced preeclampsia during a pregnancy were at significantly reduced risk of breast cancer at premenopausal ages and of HER2‐enriched breast cancer. Risk reductions amongst overall, ER positive and invasive breast cancers were observed during the 20 years since the last pregnancy with preeclampsia, with strongest reductions observed amongst premenopausal women, but not in those who last experienced preeclampsia >20 years after the pregnancy with preeclampsia. We observed no significant associations of breast cancer risk with a history of hyperemesis except for a significant positive association with HER2‐enriched breast cancer.

The literature on breast cancer risk in women with a history of preeclampsia is inconsistent. Some studies have reported an inverse association,^7^, ^8^, ^9^, ^10^, ^11^ one a positive association^12^ and others, including meta‐analyses, reported no association.^13^, ^14^, ^15^, ^16^, ^17^ Only two studies, both of case–control design, investigated the association between preeclampsia history and breast cancer by menopausal status, with one reporting a decreased risk of postmenopausal but not premenopausal breast cancer^10^ and the other no association in either group,^14^ contrary to our findings of a reduced risk of premenopausal but not postmenopausal breast cancer. One study investigated if the association may depend on age at preeclamptic pregnancy,^9^ finding no association, similar to our findings. We did not observe that the associations differed between invasive and *in situ* breast cancer, in accord with the only previous study that analysed this.^13^ The prevalence of reported preeclampsia in our study (14.8%) was higher than in clinical surveys of prevalence of this condition (e.g., 2–8%^3^). It is possible that there was over‐reporting by women including gestational hypertension i.e. hypertension in the second half of pregnancy, but without proteinuria, as preeclampsia. This potential misclassification is unlikely to have affected our results since previous studies have found the associations with breast cancer risk for gestational hypertension and preeclampsia to be similar.^10^, ^32^, ^33^

Potential mechanisms for risk reductions in breast cancer for women with a history of preeclampsia could be due to lower levels of oestrogens^4^ or of growth factors such as IGF‐1^6^ and IGF1R^34^ in such women, factors which have been implicated in breast cancer risk,^35^, ^36^ and lower levels of VEGF,^37^ an important component in breast tumour angiogenesis.^38^ Furthermore, women with preeclampsia were reported to have a lower breast cancer susceptibility polygenic risk score as well as lower mammographic density,^39^ factors which are associated with a reduced risk of breast cancer.^40^

Although there is no evidence to suggest an association between sex of the offspring and long‐term risk of breast cancer in pregnancies without complications,^41^ Vatten *et al*.^8^ and Troisi *et al*.^18^ reported that breast cancer risk is modified by offspring sex in pregnancies with preeclampsia, with strongest reductions for pregnancies involving a male foetus. Troisi *et al*.^18^ reported higher circulating levels of androgens during a pregnancy with preeclampsia with a male than a female foetus. Our results did not show such a pattern: for first pregnancies there was a significant reduction only for female offspring, although not significant heterogeneity by sex of offspring, and for last pregnancies there was no indication of an affect by sex of offspring.

Since a maternal history of preeclampsia in the pregnancy leading to the subject's birth is a risk factor for experiencing preeclampsia in the subject's own pregnancy,^42^ we investigated whether there was variation in breast cancer risk in relation to preeclampsia according to whether the subject's mother had experienced preeclampsia during the pregnancy from which the subject was born or not, but we found no significant difference between the two groups.

An adverse effect of hyperemesis on breast cancer risk has been proposed because of reported higher estradiol levels in pregnancies with hyperemesis.^43^, ^44^ We found, however, that women who had experienced hyperemesis during any of their pregnancies had similar breast cancer risk overall to women who did not, at both premenopausal and postmenopausal ages. This is contrary to results from a case–control study reporting a positive association after a recent hyperemetic pregnancy in premenopausal women^24^ and one reporting inverse associations,^14^ but accords with several other studies.^13^, ^20^, ^21^, ^22^, ^23^ The prevalence of reported hyperemesis in our study (28.7%) was much higher than in surveys of prevalence of this condition (e.g., 0.3–3%^45^, ^46^). It seems likely that there was over‐reporting by women including moderate morning sickness as hyperemesis. If any putative effects of hyperemesis on risk of breast cancer were restricted to full hyperemesis, not moderate morning sickness, then our study might have greatly underestimated any true effect. However, some studies have shown that hormone levels rise incrementally with the severity of nausea and vomiting,^43^, ^44^ suggesting that morning sickness lies at the lower range of a spectrum and hence a true effect might have been reflected to be null to some extent.

To our knowledge there have been no previous investigations of risk by tumour intrinsic subtypes of breast cancer in relation to history of preeclampsia or hyperemesis. We found that the strongest associations for both pregnancy complications were for HER2‐enriched tumours. Risk of this type of breast cancer was reduced in women with a history of preeclampsia, which is associated with lower levels of estriol,^4^ insulin‐like growth factors^5^, ^6^ and plasma epidermal growth factor levels during pregnancy,^47^ although our results were based on only 4 cases. Risk of HER2‐enriched breast cancer was significantly increased, however, in women with hyperemesis. The aetiology of HER2‐enriched breast cancer is relatively unknown, but recent studies report strong associations with the time interval between menarche and first birth,^48^ and with age at menopause.^49^

While there are several strengths to our study, including its large size, prospective design, high completeness of follow‐up and quality of covariate data, we are dependent on self‐reported history of preeclampsia and hyperemesis, although collected prior to breast cancer diagnosis and therefore unbiased by outcome. Registry‐based cohort studies have been based on records of women hospitalised for pregnancy‐complications, therefore reflecting confirmed diagnoses, but did not have complete data on other important breast cancer risk and lifestyle factors. In the Generations Study extensive pregnancy and other breast cancer risk factor data were collected and our analyses were adjusted for a wide range of potential confounders.

In conclusion, our results suggest that preeclampsia is associated with a decreased risk of breast cancer at premenopausal ages and of HER2‐enriched breast cancer. We did not find evidence that a history of hyperemesis was associated with breast cancer risk overall, but there was a positive association with the HER2‐enriched subtype.

## Supporting information

Supporting Information Table
