# Supplementary material for: Breast cancer risk in relation to history of preeclampsia and hyperemesis gravidarum: Prospective analysis in the Generations Study
Source: Int J Cancer. 2018 Mar 23;143(4):782–92. doi: 10.1002/ijc.31364 (PMC6055869; doi:10.1002/ijc.31364)
Supplement: Supplementary file 1 — Supporting Information Table [file IJC-143-782-s001.doc]

Supplementary Table 1: Relative risk of premenopausal breast cancer in relation to history of preeclampsia, overall and by invasive/*in-situ* breast cancer status.

|  | **All premenopausal breast cancer** | |  | **Premenopausal breast cancer** | | | | |
| --- | --- | --- | --- | --- | --- | --- | --- | --- |
|  |  |  |  | **Invasive** | |  | ***In-situ*** | |
| **Risk factor** | **N cases** | **Adjusted HR** (a) **(95% CI)** |  | **N cases** | **Adjusted HR** (a) **(95% CI)** |  | **N cases** | **Adjusted HR** (a) **(95% CI)** |
| *Never/ever preeclampsia* |  |  |  |  |  |  |  |  |
| Never | 480 | 1.00 (ref) |  | 404 | 1.00 (ref) |  | 76 | 1.00 (ref) |
| Ever | 48 | 0.67 (0.49-0.90) |  | 37 | 0.61 (0.43-0.86) |  | 11 | 0.96 (0.51-1.81) |
| *Number of pregnancies with preeclampsia** | | | | | | | | |
| 1 | 37 | 0.68 (0.49-0.95) |  | 28 | 0.61 (0.42-0.90) |  | 9 | 1.05 (0.52-2.10) |
| 2 | 11 | 0.74 (0.41-1.35) |  | 9 | 0.72 (0.37-1.41) |  | 2 | 0.81 (0.20-3.35) |
| ≥3 | 0 | - |  | 0 | - |  | 0 | - |
| P trend (c) |  | *0.29* |  |  | *0.57* |  |  | *0.25* |
| *Age at first preeclamptic pregnancy, years** | | | | | | | | |
| <25 | 12 | 0.83 (0.45-1.51) |  | 6 | 0.48 (0.21-1.10) |  | 6 | 2.99 (1.10-8.17) |
| ≥25 | 35 | 0.61 (0.43-0.86) |  | 31 | 0.64 (0.45-0.93) |  | 4 | 0.44 (0.16-1.20) |
| *Age at most recent preeclamptic pregnancy, years** | | | | | | | | |
| <30 | 22 | 0.60 (0.39-0.92) |  | 13 | 0.42 (0.24-0.73) |  | 9 | 1.56 (0.77-3.20) |
| ≥30 | 25 | 0.71 (0.47-1.06) |  | 24 | 0.81 (0.54-1.23) |  | 1 | 0.18 (0.02-1.30) |
| Time since *most recent* preeclamptic *pregnancy*, years*** | | | | | | | | |
| <20 | 43 | 0.64 (0.47-0.88) |  | 35 | 0.62 (0.44-0.88) |  | 8 | 0.77 (0.37-1.61) |
| ≥20 | 5 | 0.95 (0.38-2.35) |  | 2 | 0.46 (0.11-1.89) |  | 3 | 2.99 (0.84-10.69) |
| P trend (d) |  | *0.44* |  |  | *0.69* |  |  | *0.11* |
| *Preeclampsia at first pregnancy* | | | | | | | | |
| No | 486 | 1.00 (ref) |  | 409 | 1.00 (ref) |  | 77 | 1.00 (ref) |
| Yes | 42 | 0.70 (0.51-0.97) |  | 32 | 0.64 (0.44-0.92) |  | 10 | 1.05 (0.54-2.04) |
| *Preeclampsia at last pregnancy* | | | | | | | | |
| No | 510 | 1.00 (ref) |  | 426 | 1.00 (ref) |  | 84 | 1.00 (ref) |
| Yes | 18 | 0.49 (0.31-0.79) |  | 15 | 0.49 (0.29-0.82) |  | 3 | 0.49 (0.15-1.57) |
|  |  |  |  |  |  |  |  |  |

HR=hazard ratio; CI=confidence interval

*Reference group=never preeclampsia
(a) Adjusted for attained age, socio-economic score, birth cohort, benign breast disease, family history of breast cancer, age at menarche, age at first birth, number of births, duration of breast feeding, BMI at age 20 years, height, OC use, HRT use, alcohol consumption (units/wk), cigarette smoking status, physical activity level (METs/wk).

(b) Test for linear trend per pregnancy with preeclampsia, excluding zero pregnancies
(c) Test for linear trend per 5 years since last preeclamptic pregnancy

Supplementary Table 2: Relative risk of breast cancer in relation to history of preeclampsia, by history of hyperemesis gravidarum

| **Risk factor** | **Hyperemesis gravidarum** | | | | | | |
| --- | --- | --- | --- | --- | --- | --- | --- |
|  | **Never** | |  | **Ever** | |  | ***P* int** (b) |
|  | **N cases** | **Adjusted HR** (a) **(95% CI)** |  | **N cases** | **Adjusted HR** (a) **(95% CI)** |  |
| ***Preeclampsia*** |  |  |  |  |  |  |  |
|  |  |  |  |  |  |  |  |
| Never | 1,179 | 1.00 (ref) |  | 496 | 1.00 (ref) |  |  |
|  |  |  |  |  |  |  |  |
| Ever | 173 | 0.85 (0.72-0.99) |  | 121 | 0.99 (0.82-1.21) |  | 0.21 |
|  |  |  |  |  |  |  |  |

(a) Adjusted for attained age, socio-economic score, birth cohort, benign breast disease, family history of breast cancer, age at menarche, age at first birth, number of births, duration of breast feeding, menopausal status, age at menopause, BMI at age 20 years, postmenopausal BMI, height, OC use, HRT use, alcohol consumption (units/wk), cigarette smoking status, physical activity level (METs/wk).
(b) Interaction test p-value

Supplementary Table 3: Relative risk of breast cancer in relation to history of hyperemesis gravidarum, by history of preeclampsia

| **Risk factor** | **Preeclampsia** | | | | | | |
| --- | --- | --- | --- | --- | --- | --- | --- |
|  | **Never** | |  | **Ever** | |  | ***P* int** (b) |
|  | **N cases** | **Adjusted HR** (a) **(95% CI)** |  | **N cases** | **Adjusted HR** (a) **(95% CI)** |  |
| ***Hyperemesis gravidarum*** |  |  |  |  |  |  |  |
|  |  |  |  |  |  |  |  |
| Never | 1,179 | 1.00 (ref) |  | 173 | 1.00 (ref) |  |  |
|  |  |  |  |  |  |  |  |
| Ever | 496 | 1.01 (0.91-1.12) |  | 121 | 1.19 (0.94-1.50) |  | 0.21 |
|  |  |  |  |  |  |  |  |

(a) Adjusted for attained age, socio-economic score, birth cohort, benign breast disease, family history of breast cancer, age at menarche, age at first birth, number of births, duration of breast feeding, menopausal status, age at menopause, BMI at age 20 years, postmenopausal BMI, height, OC use, HRT use, alcohol consumption (units/wk), cigarette smoking status, physical activity level (METs/wk).
(b) Interaction test p-value

Supplementary Table 4: Relative risk of breast cancer in relation to history of hyperemesis gravidarum, by oestrogen receptor status and invasive/in-situ breast cancer.

|  |  | **ER status** | | | |  | **Invasive/In-situ** | | | |
| --- | --- | --- | --- | --- | --- | --- | --- | --- | --- | --- |
|  |  | **ER positive** | | **ER negative** | |  | **Invasive** | | **In-situ** | |
| **Risk factor** |  | **N cases** | **Adjusted HR** (a) **(95% CI)** | **N cases** | **Adjusted HR** (a) **(95% CI)** |  | **N cases** | **Adjusted HR** (a) **(95% CI)** | **N cases** | **Adjusted HR** (a) **(95% CI)** |
| *Never/ever hyperemesis* |  |  |  |  |  |  |  |  |  |  |
| Never |  | 1024 | 1.00 (ref) | 210 | 1.00 (ref) |  | 1144 | 1.00 (ref) | 206 | 1.00 (ref) |
| Ever |  | 467 | 1.03 (0.92-1.15) | 93 | 1.03 (0.80-1.32) |  | 519 | 1.03 (0.93-1.15) | 98 | 1.03 (0.80-1.32) |
| *Number of pregnancies with hyperemesis** | | | | | | | | | | |
| 1 |  | 173 | 0.96 (0.81-1.13) | 36 | 0.95 (0.67-1.37) |  | 197 | 0.97 (0.84-1.14) | 43 | 1.15 (0.82-1.61) |
| 2 |  | 214 | 1.12 (0.96-1.30) | 39 | 1.04 (0.73-1.49) |  | 233 | 1.10 (0.95-1.27) | 41 | 1.05 (0.74-1.48) |
| ≥3 |  | 80 | 0.98 (0.76-1.25) | 18 | 1.18 (0.69-2.00) |  | 89 | 1.01 (0.80-1.28) | 14 | 0.73 (0.41-1.29) |
| P trend (c) |  |  | *0.85* |  | *0.64* |  |  | *0.96* |  | *0.07* |
| *Age at first hyperemetic pregnancy, years** | | | | | | | | | | |
| <25 |  | 197 | 1.06 (0.88-1.28) | 46 | 1.20 (0.81-1.76) |  | 223 | 1.09 (0.91-1.29) | 43 | 1.00 (0.68-1.48) |
| ≥25 |  | 270 | 1.01 (0.88-1.16) | 47 | 0.93 (0.67-1.28) |  | 296 | 1.01 (0.88-1.15) | 55 | 1.04 (0.77-1.42) |
| *Age at most recent hyperemetic pregnancy, years** | | | | | | | | | | |
| <30 |  | 279 | 1.00 (0.87-1.15) | 57 | 1.00 (0.73-1.36) |  | 315 | 1.02 (0.89-1.16) | 62 | 1.05 (0.78-1.43) |
| ≥30 |  | 188 | 1.07 (0.91-1.25) | 36 | 1.07 (0.75-1.53) |  | 204 | 1.06 (0.91-1.23) | 36 | 0.99 (0.69-1.42) |
| Time since most recent *hyperemetic pregnancy*, years* | | | | | | | | | | |
| <20 |  | 153 | 1.12 (0.94-1.34) | 26 | 0.81 (0.53-1.24) |  | 162 | 1.05 (0.88-1.25) | 35 | 1.08 (0.73-1.57) |
| ≥20 |  | 314 | 0.98 (0.85-1.12) | 67 | 1.16 (0.86-1.57) |  | 357 | 1.02 (0.90-1.16) | 63 | 1.00 (0.73-1.36) |
| P trend (d) |  |  | *0.77* |  | *0.51* |  |  | *0.81* |  | *0.30* |
| *Hyperemesis at first pregnancy* | | | | | | | | | | |
| No |  | 1067 | 1.00 (ref) | 221 | 1.00 (ref) |  | 1195 | 1.00 (ref) | 218 | 1.00 (ref) |
| Yes |  | 424 | 1.05 (0.94-1.18) | 82 | 1.01 (0.78-1.31) |  | 468 | 1.05 (0.94-1.17) | 86 | 1.01 (0.78-1.30) |
| *Hyperemesis at last pregnancy* | | | | | | | | | | |
| No |  | 1142 | 1.00 (ref) | 230 | 1.00 (ref) |  | 1273 | 1.00 (ref) | 236 | 1.00 (ref) |
| Yes |  | 349 | 1.01 (0.90-1.14) | 73 | 1.07 (0.82-1.40) |  | 390 | 1.02 (0.91-1.15) | 68 | 0.91 (0.69-1.20) |
|  |  |  |  |  |  |  |  |  |  |  |

ER= oestrogen receptor; HR=hazard ratio; CI=confidence interval

*Reference group=never hyperemesis
(a) Adjusted for attained age, socio-economic score, birth cohort, benign breast disease, family history of breast cancer, age at menarche, age at first birth, number of births, duration of breast feeding, menopausal status, age at menopause, BMI at age 20 years, postmenopausal BMI, height, OC use, HRT use, alcohol consumption (units/wk), cigarette smoking status, physical activity level (METs/wk).
(b) Test for linear trend per pregnancy with hyperemesis, excluding zero pregnancies
(c) Test for linear trend per 5 years since last hyperemetic pregnancy
